# Regulation of Telomere Homeostasis during Epstein-Barr virus Infection and Immortalization

**DOI:** 10.3390/v9080217

**Published:** 2017-08-09

**Authors:** Siamak A. Kamranvar, Maria G. Masucci

**Affiliations:** 1 Department of Medical Biochemistry and Microbiology, Biomedical Center, Uppsala University, 751 23 Uppsala, Sweden; Siamak.kamranvar@imbim.uu.se; 2 Department of Cell and Molecular Biology, Karolinska Institutet, 171 77 Stockholm, Sweden

**Keywords:** telomere, EBV, telomerase, ALT

## Abstract

The acquisition of unlimited proliferative potential is dependent on the activation of mechanisms for telomere maintenance, which counteracts telomere shortening and the consequent triggering of the DNA damage response, cell cycle arrest, and apoptosis. The capacity of Epstein Barr virus (EBV) to infect B-lymphocytes in vitro and transform the infected cells into autonomously proliferating immortal cell lines underlies the association of this human gamma-herpesvirus with a broad variety of lymphoid and epithelial cell malignancies. Current evidence suggests that both telomerase-dependent and -independent pathways of telomere elongation are activated in the infected cells during the early and late phases of virus-induced immortalization. Here we review the interaction of EBV with different components of the telomere maintenance machinery and the mechanisms by which the virus regulates telomere homeostasis in proliferating cells. We also discuss how these viral strategies may contribute to malignant transformation.

## 1. Introduction

Epstein-Barr virus (EBV) is a ubiquitous gamma-herpesvirus that establishes life-long persistent infections in the vast majority of humans [1]. Primary infection is usually asymptomatic but, when delayed until adolescence or adulthood, it may cause a benign lymphoproliferative disease known as infectious mononucleosis [2]. B-lymphocytes are the site of EBV persistence in vivo, although different types of epithelial cells and T or Natural Killer (NK) cells, may also be infected. Despite its widespread diffusion and apparent harmlessness, EBV is causally linked to a broad spectrum of malignancies of lymphoid and epithelial cell origin, including Burkitt’s lymphoma (BL), Hodgkin’s lymphoma (HD), post-transplant and AIDS-associated lymphomas, nasopharyngeal (NPC), and a subset of gastric carcinoma [3].

The oncogenic potential of EBV is epitomized by its capacity to immortalize primary B-lymphocytes in vitro, giving rise to autonomously growing Lymphoblastoid Cell Lines (LCLs). Growth transformation is achieved through the expression of few latency-associated viral genes that encode proteins—six EBV nuclear antigens (EBNA1-6, also known as EBNA1, -2, -3A, 3B, 3C, and –leader protein or LP) and three latent membrane proteins (LMP-1, LMP-2A, LMP-2B), small non-polyadenylated non-coding double-stranded RNAs (EBERs), and microRNAs [4,5]. Studies carried out with recombinant EBV strains lacking individual latency genes have elucidated the contribution of these viral products to B-cell growth transformation. EBNA-2 and LMP-1 are essential for the initiation and maintenance of cell proliferation, whereas EBNA-1, -3, -5, and -6 play crucial complementary roles [3]. Thus, the concerted action of several EBV products derange signaling pathways controlling cell growth and survival. However, expression of the viral latency gene products is not sufficient for full immortalization of the infected cells. Only cells capable of maintaining telomere integrity through unlimited numbers of cell divisions can escape cellular senescence and become truly immortal [6,7]. The observation that the majority of EBV-driven tumors are telomerase-positive supports the relevance of telomere maintenance in the process of tumorigenesis. Before reviewing the strategies used by EBV to interfere with telomere homeostasis, we will briefly summarize our current understanding of how different pathways of telomere maintenance contribute to cell immortalization and oncogenesis.

## 2. Telomere Maintenance and Cell Immortalization

**Figure 1:**
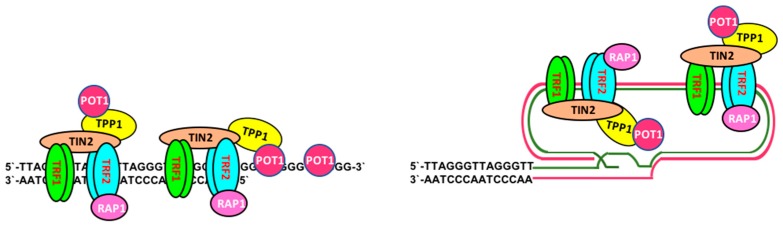
Schematic illustration of the structure of telomeres. The telomere repeats are coated by shelterin, a complex of six proteins that bind to DNA either directly (TRF1, TRF2, and POT1) or indirectly (TIN2, RAP1, and TPP1). The shelterin induces the formation of a T-loop by invasion of the single-stranded overhang into double-stranded telomeric DNA. TRF: telomeric repeat-binding factor; POT1: protection of telomeres 1; TIN2: TRF1-interacting nuclear factor 2; RAP1: repressor-activator protein 1.

The telomeres are nucleoprotein complexes that cap the ends of linear chromosomes, which prevents triggering of the DNA-damage response by naked linear DNA, and controls the cellular proliferative lifespan [8]. Each telomere is composed of a long double-stranded repeat array of the 5′-TTAGGG-3′ nucleotide sequence coated with a protein complex, named shelterin, that is formed by the DNA binding proteins telomeric repeat-binding factor 1 (TRF1), TRF2, and protection of telomeres 1 (POT1), and their binding partners, TRF1-interacting nuclear factor 2 (TIN2), repressor- activator protein 1 (RAP1), and TPP1. RAP1 interacts with TRF2, TIN2 connects TRF1 to TRF2 and binds to TPP1 that is, in turn, an interacting partner of POT1 [9]. TRF1 and TRF2 bind to double-stranded telomeric DNA, whereas POT1 is associated with a short single-stranded G-rich 3′-overhang whose invasion into the double-stranded telomeric DNA forms a T-loop at the end of telomeres (Figure 1) [10]. The loop structure stabilized by the shelterin complex shields the chromosome ends from recognition by the DNA damage response (DDR) machinery and degrading enzymes [10]. Depletion of the shelterin proteins causes telomere de-protection and the local accumulation of components of the DNA damage response, including meiotic recombination 11 homolog (Mre11), phosphorylated histone 2AX (γH2AX), and p53 binding protein 1 (53BP1), that assemble into complexes known as Telomere dysfunction Induced Foci (TIFs) [11]. Depletion of TRF2 activates ATM-dependent DNA double-strand break repair, whereas POT1 prevents the activation of ATR-mediated repair at telomeric single-stranded DNA [11,12]. The TRF2-RAP1 complex prevents the engagement of telomeres in homologous recombination-mediated deletions and fusion [13]. Beside its role in telomere protection, the shelterin complex is also involved in the regulation of telomere length. Thus, overexpression of TRF1 and TRF2 results in rapid telomere shortening [14]. In addition to protection by shelterin, other mechanisms contribute to maintain the integrity of telomeres. The G-rich telomeric sequence may promote the formation of stable DNA structures, such as G-quadruplexes, that are resistant to nucleolytic degradation [15]. The telomeric transcript, a small non-coding Telomeric Repeat-containing RNA (TERRA), binds to TRF2 and promotes the recruitment of the Origin Recognition Complex (ORC), which contributes to telomere maintenance through the formation of heterochromatin [16].

Telomere length restricts the replicative lifespan of somatic cells because telomere repeats are lost at each cell division due to inability of the DNA polymerase to faithfully replicate the 3′-end of linear DNA [17]. Thus, after a certain number of cell divisions, the shortened telomeres become unable to maintain the protective structure and, similar to DNA breaks, trigger the DDR, which leads to cell cycle arrest and replicative senescence or apoptosis [18]. This process may be activated in somatic cells by a single critically short telomere [19]. In malignant cells, the telomere-mediated proliferation barrier is bypassed through the activation of compensatory mechanisms that preserve the length of telomeres. Most tumors prevent telomere shortening by activating telomerase, a reverse transcriptase enzyme that is silenced in somatic cells [20], whereas 10–15% of tumors utilize a telomerase-independent mechanism named Alternative Lengthening of Telomere (ALT) [21].

### 2.1. Telomerase-Dependent Telomere Maintenance

**Figure 2:**
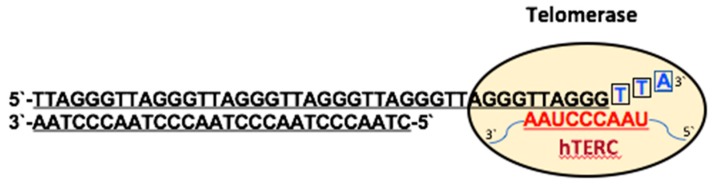
Schematic illustration of telomerase-dependent telomere elongation. Few nucleotides of the G-rich 3′ overhang pair with their complementary sequence in the telomerase RNA (hTERC) and the reverse transcriptase activity of hTERT extends the telomere using the hTERC as a template. The extended DNA terminus detaches from its RNA template, becoming available for another round of elongation by telomerase.

The telomerase is a ribonucleoprotein complex containing a catalytic subunit, human telomerase reverse transcriptase (hTERT), and an RNA template, human telomerase RNA component (hTERC), that are essential for activity of the enzyme [22]. The reverse transcriptase hTERT adds GGTTAG repeats to the 3′-overhang of telomeres using hTERC as an internal template (Figure 2) [23]. The telomerase inserts several telomeric repeats before detachment from the chromosome in a shelterin-regulated process [24]. TIN2 promotes the nuclear import of TPP1 and POT1, whereas TPP1 controls their nuclear abundance through its nuclear export signal. Protein abundance controls the nuclear assembly of the shelterin complex, which in turn regulates the access of telomerase to the telomere ends [25].

The activity of telomerase is inhibited during cellular differentiation and hTERT becomes undetectable in most normal human somatic cells, except stem cells and lymphocytes [26,27,28]. In malignant cells the enzyme is activated by a variety of different mechanisms [29]. Beside genetic amplification of the *hTERT* locus, epigenetic deregulation, point mutations within the telomerase promoter, and post-translational modifications can regulate the expression, nuclear translocation, and enzymatic activity of telomerase. The hTERT promoter contains binding sites for c-Myc and Sp1 [30,31], and activating mutations often create additional binding sites for the GABP transcription factor [32,33]. C-Myc interacts with the transcriptional coactivator TRRAP, which induces acetylation of histones H3 and H4 at the hTERT promoter and activates transcription [34]. The mitogen-activated protein kinase (MAPK) signaling pathway regulates the expression of hTERT in response to different stimuli via phosphorylation of histone H3 at the hTERT promoter, which promotes histone acetyltransferases (HATs)-mediated acetylation on Lys14 [35]. This enhancing effect is counteracted by histone deacetylases (HDACs) [36]. Protein kinase C (PKC) and protein kinase B (AKT) mediate phosphorylation and nuclear translocation of hTERT, whereas protein phosphatase-2A (PP2A) dephosphorylates hTERT and inhibits telomerase activity [37,38]. The RelA/p65 subunit of nuclear factor-κB (NF-κB) binds to hTERT and facilitates its translocation to the nucleus [39,40]. AP-1 represses hTERT transcription via Jun-D and c-Jun promoter binding sites in some cancer cells, whereas the c-Jun N-terminal kinase (JNK) increases hTERT gene expression [41,42]. Several other proteins such as mitotic arrest deficient protein 1 (Mad-1), transforming growth factor-β (TGF-β), upstream stimulatory factor 1 (USF), breast cancer type 1 susceptibility protein (BRCA1), PIN2/TRF1-interacting telomerase inhibitor 1 (PinX1), NFX1-91, and p53 are negative regulators of hTERT [43,44,45,46,47,48,49]. The TERRA transcript may affect telomere maintenance via direct inhibition of telomerase activity or by promoting exonuclease 1-mediated resection of the telomere ends [50,51].

### 2.2. Telomerase-Independent Telomere Maintenance (ALT)

**Figure 3:**
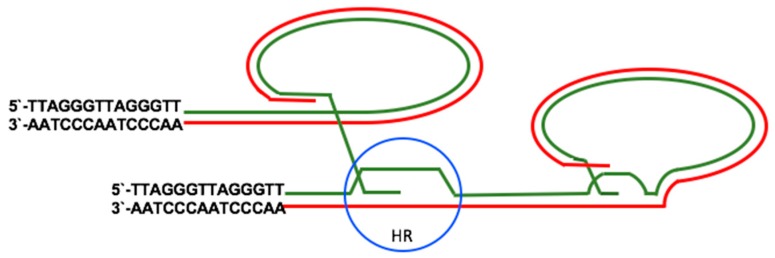
Schematic illustration of ALT-mediated telomere elongation. In ALT, Holliday-junctions (HJ) are formed between two sister telomeres (blue circle) or between one telomere and extra-telomeric DNA and the telomeres are elongated by homologous recombination (HR). This reaction often occurs inside promyelocytic leukemia nuclear bodies (PML-NBs).

Cancers that lack telomerase activity exploit a DNA repair-based mechanism for telomere elongation referred to as ALT [52]. Characteristic features of ALT include telomere length heterogeneity, ranging from very short to longer than 50 kb, the presence of extrachromosomal telomeric repeats (ECTRs), increased rate of telomere sister chromatid exchange (TSCEs), mutations of the chromatin remodeler ATRX, and genomic instability [53]. Proteins involved in homologous recombination (HR), such as components of the Mre11-Rad50-Nbs1 (MRN) complex, RAD51, structural maintenance of chromosomes (SMC)-5 and -6, E3 SUMO-protein ligase MMS21, flap endonuclease 1 (FEN1), crossover junction endonuclease MUS81, FANCA, and FANCD2, are required for ALT [54,55,56]. The HR proteins gather, together with telomeric DNA and telomere-binding proteins, into promyelocytic leukemia (PML) nuclear bodies (PML-NBs) to form ALT-associated PML Bodies (APBs), a distinctive feature of ALT cells [56]. PML-NBs contain, in addition to PML, SP100, ATRX, and DAXX that are involved in chromatin assembly and regulation. ATRX is a histone H3.3 specific chaperone containing SNF-like ATPase remodeling activity, while DAXX recruits HDACs [57]. The first step in ALT is the invasion of the single stranded 3’-overhang into a telomeric DNA template, which is normally inhibited by the shelterin complex [58]. RAD51 promotes ALT by inducing the displacement of replication protein A (RPA) from the single stranded overhang [59]. The second step, template-directed synthesis of telomeric DNA, is followed by a third step in which the intermediate products of HR are processed as Holliday-junctions (HJ)-dissolution and HJ-resolution leading to lower and higher rate of telomere sister chromatid exchanges, respectively (Figure 3) [60].

Although the involvement of DDR proteins suggests that DNA damage at telomeres is a major cause of ALT activation [58,61,62,63], changes in chromatin architecture are likely to play important roles. The telomeres of ALT cells exhibit lower nucleosome density and decreased mobility compared to healthy telomeres [64,65]. While the formation of telomeric heterochromatin is associated with trimethylation of histone H3K9, trimethylation of histone H4K20, binding of Heterochromatin Protein 1 (HP1) and histone hypoacetylation, all of which suppress telomeric recombination [66], loss of ATRX, or its binding partner DAXX, promote ALT [67]. Indeed, telomeric DNA damage, disruption of the ATRX/DAXX complex or inhibition of telomerase activity were found to be equally powerful inducers of a switch to ALT in telomerase-positive cancer cells [68]. In addition, telomere sequence alterations may also promote ALT by facilitating the recruitment of HR proteins or other regulators. These include for example the nuclear hormone receptor protein TR4 and COUP transcription factor 2 (COUP-TF2) that bind to zinc finger protein ZNF827, which in turn recruits the chromatin remodeling complex nucleosome remodeling deacetylase (NuRD). The NuRD–ZNF827 complex promotes telomere de-compaction and the recruitment of DDR proteins at telomeres [69,70].

## 3. Telomere Maintenance in EBV-Infected Cells

**Figure 4:**
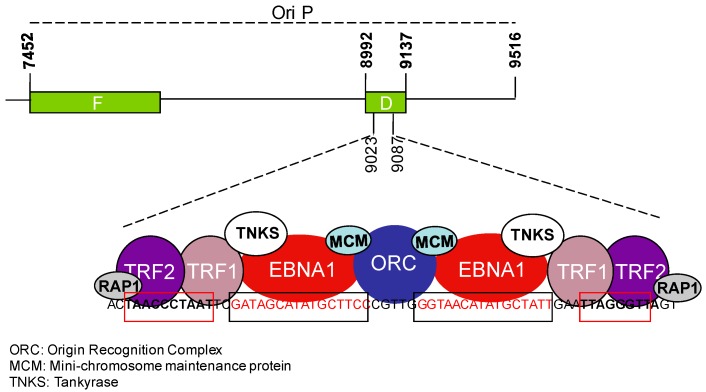
Schematic illustration of the Epstein Barr virus (EBV) OriP and interacting viral and telomeric proteins. Organization of the OriP region where the family of repeats (F) and dyad symmetry (DS) regions are indicated. The enlarged region within the DS contains two EBV nuclear antigen (EBNA)1 binding sites (black box) adjacent to telomere-like sequences (red box) where EBNA1 and telomere-associated proteins bind and recruit proteins, such as Origin Recognition Complex (ORC) and MCM, that are involved in viral genome replication and maintenance. TRF1-interacting protein, TNKS, interacts with EBNA1 and regulates OriP replication.

Tumor viruses must override the telomere erosion barrier in order to achieve unlimited proliferation of the infected cells [71]. A corollary of this effect is the capacity of many DNA viruses to harness features and components of the telomere maintenance machinery to promote their own persistence and replication. Most DNA viruses utilize telomere-like structures, called terminal repeats (TRs), to stabilize their linear genomes, suggesting that viral-like elements may be the evolutionary precursors of cellular telomeres [72,73]. The TRs mediate circularization of the linear viral DNA, which is required for persistence of the virus as a nuclear episome [73]. The circularization of herpesvirus DNA depends on DNA Ligase IV and XRCC4 that are the main components of the non-homologous end-joining repair machinery [74]. In addition to the TRs, the EBV genome comprises an internal repeat element, the origin of plasmid replication, OriP, that includes the family of repeats (FRs) and dyad symmetry (DS) elements [75]. The viral episome maintenance protein EBNA1 and the shelterin proteins TRF1 and TRF2 bind to OriP (Figure 4). TRF2 recruits ORC to the DS, which facilitates DNA replication and episome maintenance [76].

Another feature shared by sub-telomeric chromatin and the EBV genome is the presence of binding sites for the CCCTC-binding factor (CTCF) and cohesin [77,78]. In cellular telomeres, CTCF regulates the transcription of TERRA and the binding of cohesin and RNA polymerase II (RNAPII) to the sub-telomeric region. Depletion of either CTCF or cohesin causes the formation of TIFs, and destabilizes the binding of TRF1 and TRF2 to telomeric DNA [79]. CTCF, cohesin, and RNA Polymerase II were shown to regulate the transcription of several DNA viruses [80]. In EBV, binding of CTCF to sequences located between OriP and the RBP-Jκ response elements of the C promoter (Cp) regulates the transcription levels of EBNA2 and plays an important role in the control of latency [77].

The EBV genome maintenance protein EBNA1 shares structural and functional similarity with shelterin subunits and with other telomere associated proteins. EBNA1 and TRF2 contain RGG-like motifs that mediate the tethering of EBNA1 to metaphase chromosome and are involved in the recruitment of ORC [81,82,83]. Both EBNA1 and TRF2 bind TERRA in vitro but only TRF2 does so efficiently in cells, whereas EBNA1 and TRF1 interact with the EBV non-coding RNAs, EBERs [16,84]. EBNA1 binds to and is a substrate of Tankyrase, a poly-ADP ribosylase that also binds to TRF1. Poly-ADP ribosylation modifies the binding and function of EBNA1 at OriP [85]. The stalling of replication forks induced by EBNA1 recruits the replisome protection factor Timeless that has a function in sister chromatid cohesion and telomere length maintenance [86,87,88]. Finally, telomere associated cellular proteins play direct or indirect roles in the regulation of EBV gene expression. The capacity of ATRX to repress TERRA transcription at telomeres is mirrored by its inhibitory effect on viral transcription and replication [89,90]. hTERT may also cooperate with EBNA2 in the inhibition EBV lytic replication via induction of BATF, a transcription factor activated by neurogenic locus notch homolog protein 2 (NOTCH2) that negatively regulates the expression of an immediate-early viral gene *BZLF1*, the master switch of the viral lytic cycle [91,92]. hTERT transactivates the NOTCH2 promoter via NF-κB signaling [92] while EBNA2 mimics NOTCH2 signaling and induces BATF expression early after infection of primary B-cells, which may play a key role in the establishment of latency [93].

### 3.1. EBV and the Regulation of Telomerase Activity

EBV-associated tumors provide the only example of malignancies where the telomeres of malignant virus infected cells are longer compared to the corresponding uninfected malignant or non-malignant cells [94]. The average length of telomeres was shown to remain constant or even increase during the early phases of EBV-induced growth transformation of primary B-lymphocytes [95,96,97], and EBV-positive BL lines, such as Namalwa, Raji, and EB-3, were shown to have longer telomeres compared to EBV-negative BLs [98]. Interestingly, short telomeres were also observed in the EBV positive BL line Daudi that carries a transformation-defective EBV strain [98]. Transcriptional activation of hTERT is an important mechanism by which EBV contributes to telomere maintenance. Several viral latency proteins were shown to have positive or negative effects on the expression of telomerase. The latent membrane protein LMP1 increases telomerase activity through induction of the NF-κB, MAPK, and extracellular signal-regulated kinases (ERK1/2) pathways, which promote the transcriptional activation of hTERT [99]. LMP1 also induces c-Myc-mediated trans-activation of the hTERT promoter in primary human nasopharyngeal epithelial cells and in a nasopharyngeal carcinoma cell line, while inhibition of LMP1 is associated with telomerase downregulation and induction of apoptosis in EBV-positive lymphomas [100,101]. The constitutive activation of the MAPK pathway in LMP1 expressing cells suggests that MAPK-mediated control of H3 phosphorylation may play an additional role in the regulation of hTERT expression [102]. In addition, LMP1 may functionally activate telomerase by promoting the p65-dependent nuclear translocation of hTERT [103]. In contrast, the other EBV-encoded latency-associated membrane protein, LMP2A, inhibits telomerase activity through its tyrosine-based activation motif, ITAM, which is involved in the regulation of B-cell activation and inhibits the transition from latent to productive infection [104]. Recruitment of the Syk tyrosine kinase to the LMP2A ITAM motifs induces ERK signaling, which leads to AP1-mediated repression of hTERT transcription [104]. The nuclear protein EBNA2 is also involved in the regulation of hTERT through its capacity to transactivate both LMP1 and LMP2A and stimulate the expression of cellular genes, such as the *c-myc* proto-oncogene [105]. EBNA2 does not bind directly to DNA but activates the transcription of various genes through hijacking the DNA-binding protein CBF1 that recruits a transcription complex containing p300, CREB-binding protein (CBP), and P300/CBP-associated factor (P/CAF) histone acetyltransferases [106].

### 3.2. EBV and the Activation of ALT

Morphological analysis of telomeres in EBV-carrying tumor cell lines and EBV-infected primary B-lymphocytes suggests that infection is associated with the activation of ALT. A remarkable heterogeneity of telomere size was observed by Fluorescent In Situ Hybridization (FISH) in EBV-carrying BL cell lines compared to their EBV-negative counterparts [107]. Expression of EBNA1 alone was shown to be sufficient for the induction of several ALT associated traits, including the occurrence of ECTRs, telomere length heterogeneity, and enhanced telomere sister chromatid exchanges [107,108]. Telomere dysfunction is an early effect of EBV infection in primary B-cells [97]. It is noteworthy that pre-immortal EBV-infected B-lymphocytes show no or very low telomerase activity and may undergo more than 150 population-doublings before strong telomerase activity becomes detectable, which usually correlates with recovery from growth crisis, decreased signs of genomic instability and immortalization [6]. The remarkable increase of ECTRs in growth transformed cell lines that express no or low levels of telomerase [95,97], suggests that ECTR DNA may serve as template for ALT.

Several mechanisms may contribute to the activation of ALT in EBV-infected B-lymphocytes. The EBV major tegument protein BNRF1 interacts with DAXX at PML-NBs and disrupts the formation of the DAXX–ATRX complex, which plays a supporting role in EBV primary infection [109]. Interestingly, delivery of the BNRF1 protein in virus-like particles was shown to promote centrosome amplification and chromosomal instability, which may confer an increased risk of malignant transformation [110]. We and others have shown that the early phase of infection is associated with telomere de-protection, which can trigger the DDR, leading to the activation of HR or non-homologous end joining (NHEJ) at telomeres [95,97]. This is likely to be the primary cause of chromosomal instability that is manifested as a remarkably high frequency of non-clonal structural aberrations, unbalanced translocations, and chromatid gaps soon after infection. EBV-infected primary B-cells express significantly lower levels of TRF1, TRF2, POT1, and ATRX, compared to established LCLs [97], which may be partly due to the capacity of LMP1 to downregulate several shelterin subunits, including TRF1, TRF2, and POT1 [111]. Both EBV-infected primary B-lymphocytes and EBV-carrying BL lines show a relative depletion of TRF2 at telomeres [97,107]. In BL cells, the occurrence of telomere damage and the displacement of TRF2 were shown to be directly correlated with the induction of oxidative stress, which is mediated by EBNA1 via transcriptional regulation of the catalytic subunit of the nicotinamide adenine dinucleotide phosphate (NADPH) oxidase Nox2 and consequent accumulation of reactive oxygen species (ROS) [97,112]. Oxidative stress may cause telomere de-protection via induction of multiple 8-oxo-guanine lesions, abasic sites, and single nucleotide gaps that hamper the binding of TRF1 and TRF2 to telomeric DNA [113]. The activation of ALT in response to the irreparable damage caused by the oxidation of telomeric DNA [114] may prevent the oxidative damage from reaching levels that threaten cell survival [115]. Sustained accumulation of ROS was observed also in primary EBV-infected B-lymphocytes where it correlates with high levels of DNA damage and activation of the DDR [97]. The high levels of ROS are likely to play a key role in growth transformation since treatment with ROS scavengers is associated with severe inhibition of cell proliferation, downregulation of the viral LMP1, and decreased phosphorylation of the cellular transcription factor STAT3 [116]. Collectively, these observations suggest that the high levels of ROS induced by EBV infection may promote the activation of ALT via two complementary mechanisms: by induction of irreparable DNA breaks in the G-rich telomeric repeats, which forces the activation of recombination-based repair [117], and by promoting functional inactivation of the shelterin complex due to inefficient binding of TRF1 and TRF2 to oxidized telomeric DNA.

## 4. Conclusions

The human tumor viruses have evolved elaborate strategies to bypass the numerous constraints that prevent uncontrolled cell proliferation. Virus-induced growth transformation is often accompanied by the induction of telomere dysfunction, which is in turn associated with DNA damage and genomic instability [94]. The resulting destabilization of the cellular genome is likely to play an important role in the transition of the infected cells to full malignancy but is also associated with activation of the DDR and cell senescence. Thus, immortalization requires the concerted activity of functions that dampen the growth inhibitory effects of telomere dysfunction and allow the maintenance of relatively functional telomeres.

EBV provides a clear example of the complex relationship between a tumor virus and the telomeres. On the one side, the functional and structural similarity between the EBV genome and cellular telomere is epitomized by the direct or indirect binding of several members of the shelterin complex to viral DNA where they play important roles in virus persistence and replication. It is tempting to speculate that the retargeting of shelterin and other telomere regulatory components to the viral genome could be partly responsible for the telomere de-protection observed in freshly EBV-infected B-lymphocytes. The oxidative environment that characterizes the early phase of EBV-induced growth transformation could also cause telomere de-protection via oxidation-dependent inhibition of the binding of TRF1 and TRF2 to telomeric DNA. It is noteworthy that, while potentially dangerous for the cell, the induction of high levels of ROS appears to be essential for growth transformation, possibly via remodeling of the cellular and viral microRNA (miRNA) transcription landscape [116]. Oxidative stress is also associated with the induction of irreparable DNA damage at telomeres, which, together with telomere de-protection, is likely to be a major drive in the activation of ALT. In addition, the failure to activate robust telomerase activity soon after infection could also provide a strong stimulus for ALT activation. ALT is mediated by DNA repair mechanisms that are intrinsically imprecise, which may contribute to chromosome and genomic instability. Virus-induced telomere dysfunction and genomic instability could play an important role in tumor progression by promoting the occurrence of mutations that may ultimately lead to the selection of highly malignant cells.

**Figure 5:**
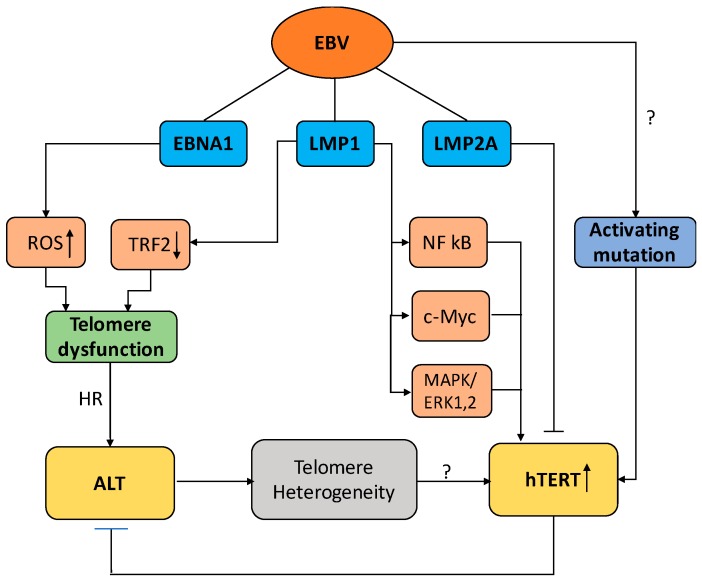
Cartoon illustrating different pathways of viral interference with telomere maintenance during EBV-induced B-cell immortalization. Latent membrane protein 1 (LMP1) promotes the activation of telomerase through several pathways that regulate the transcription and nuclear translocation of human telomerase reverse transcriptase (hTERT), whereas LMP2A inhibits telomerase activity by transcriptional repression of hTERT. Activating mutation in the promoter region of hTERT may contribute to the capacity of EBV to regulate the activity of telomerase. EBNA1 and LMP1 promote telomere dysfunction by inducing oxidative stress and downregulation of TRF2, respectively. Telomere dysfunction in the absence of telomerase activity activates ALT in which telomeres homeostasis is maintained by HR, which results in telomere length heterogeneity. The generation of few extremely short telomeres may, together with the occurrence of activating mutations, contribute to the activation of telomerase.

While the activation of ALT could sustain long-term cell proliferation, the activation of telomerase provides a more efficient mean to assure telomere integrity and cellular immortality. Indeed, telomerase is upregulated in the vast majority of EBV-carrying tumors in vivo, and several EBV products were shown to regulate the activity of telomerase in established LCLs. In this context, it is somewhat surprising that strong telomerase activity is detected only weeks or months after infection of primary B-cells, while the latency-associated program of viral gene expression is usually established within the first few days of infection. There are two non-mutually exclusive possible explanations for this finding. The level of telomerase activity may be determined by the balance between the opposite effects of viral products that are expressed at different levels during the early and late phases of cell immortalization. Alternatively, it is also possible that the capacity of viral proteins to efficiently activate telomerase may be dependent on the occurrence of activating mutations in the hTERT promoter. Such mutations are relatively frequent in certain types of human cancers [32], but their occurrence in EBV-associated tumors has not been investigated (Figure 5).

In conclusion, the findings summarized in this review suggest a complex scenario of EBV oncogenesis where the capacity of viral products to drive cell proliferation in the absence of telomerase activity may promote the activation of recombination-based mechanisms for telomere homeostasis that are inherently imprecise and could both initiate and propagate genetic alterations that drive oncogenesis. The activation of telomerase may contribute to tumor progression by allowing the selection of clones with inheritable genetic alterations capable of conferring significant growth advantages. Further investigation in this area will certainly yield better insights on the mechanism of EBV oncogenesis and may suggest novel therapeutic approaches for the human cancers associated with this virus infection.
